# Sensitivity of *Staphylococcus aureus* cultures of different biological origin to commercial bacteriophages and phages of *Staphylococcus aureus* var*. bovis*

**DOI:** 10.14202/vetworld.2021.1588-1593

**Published:** 2021-06-19

**Authors:** Yulia Horiuk, Mykola Kukhtyn, Serhiy Kernychnyi, Svitlana Laiter-Moskaliuk, Sergiy Prosyanyi, Nataliia Boltyk

**Affiliations:** 1 Department of Infectious and Parasitic Diseases, State Agrarian and Engineering University in Podilya, Kamianets-Podilskyi, Ukraine; 2 Department of Food Biotechnology and Chemistry, Ternopil Ivan Pului National Technical University, Ternopil, Ukraine; 3 Department of Veterinary Obstetrics, Pathology and Surgery, State Agrarian and Engineering University in Podilya, Kamianets-Podilskyi, Ukraine; 4 Department of Animal Hygiene and Veterinary Support of the Cynological Service of the National Police of Ukraine, State Agrarian and Engineering University in Podilia, Kamianets-Podilskyi, Ukraine; 5 Research Station of the Institute of Veterinary Medicine of NAAS, Ternopil, Ukraine

**Keywords:** bacteriophage, drug development, mastitis, *Staphylococcus aureus*

## Abstract

**Background and Aim:**

Mastitis, an inflammation of the mammary gland, is an ongoing problem in dairy herds. In this study, we determined the sensitivity of *Staphylococcus aureus* cultures of different biological origins to commercial bacteriophages and phages of *S. aureus* var*. bovis* which were isolated on dairy farms, to create a drug for the treatment of mastitis in cows.

**Materials and Methods:**

We used cultures of *S. aureus* isolated from different habitats, and other types of staphylococci isolated on dairy farms. As antibacterial agents, the commercially available bacteriophages staphylococcal bacteriophage and Intestifag and field strains of phages *Phage SAvB07*, *Phage SAvB08*, *Phage SAvB12*, and *Phage SAvB14* were used. Evaluation of their lytic properties was performed using the drip method.

**Results:**

The drug Intestifag lysed cultures isolated from human habitats and archival strains of *S. aureus* No.209-P and *S. aureus* (ATCC 25923) in 91.8%–100% of cases. Staphylococcal bacteriophage killed 3.6 times fewer cultures of *S. aureus* isolated from humans than Intestifag and did not affect the growth of archival strains. Neither drug lysed cultures isolated from cows or cultures isolated from dairy products sold in agri-food markets. *Phage SAvB14* lysed 92.7±8.3% of *S. aureus* isolated from the mammary glands of cows and 69.2±6.4% of cultures isolated from dairy products sold in agri-food markets. *Phage SAvB12*, *Phage SAvB08*, and *Phage SAvB07* lysed 1.2-1.7 times fewer cultures isolated from the mammary glands of cows and 6-18 times fewer cultures isolated from dairy products, compared with *Phage SAvB14*. Phages of *S. aureus* var*. bovis* can infect staphylococcal species such as *Staphylococcus epidermidis, Staphylococcus haemolyticus*, *Staphylococcus saprophyticus*, and *Staphylococcus xylosus*. The widest range of hosts was found for *Phage SAvB14*, which indicates its polyvalence.

**Conclusion:**

The biological origin of staphylococcal strains must be considered when developing effective phage therapy. *Phage SAvB14* appears to be a good candidate for the development of a drug for the treatment of mastitis in cows.

## Introduction

The increasing spread of antibiotic resistance among clinical isolates of *Staphylococcus aureus* is a major problem in human and veterinary medicine [1-3]. The genus *Staphylococcus* includes commensals and pathogens in both humans and animals [4]. Diseases caused by staphylococci, range from common food poisonings to serious nosocomial and livestock infections, including mastitis and endometritis [5,6]. As a result of the high frequency of carriers of staphylococci, the variety of pathologies caused by these bacteria, and the lack of an effective licensed vaccine, research into the development of new drugs against staphylococci is becoming increasingly important [7,8].

Bacteriophages are viruses that reproduce only in prokaryotic cells [9]. These very host-specific agents are ubiquitous and can be isolated from the environment with relatively little effort and time. Phage binds to outer membrane proteins, lipopolysaccharides, or components of the bacterial capsules, pili, or flagella on the surface of the host cell. The phage then inserts its genome into the bacterial cell. After replication of phage DNA or RNA, phage-encoded enzymes lead to lysis of the bacterial cell, releasing next-generation phages; the virus population grows exponentially [10,11]. The proliferation of phages occurs as long as there is a sufficiently high concentration of bacteria in the immediate area. This characteristic is one of the main advantages of phage therapy. There are several ways in which bacteriophages can be classified, but for therapeutic purposes, researchers generally categorize phages as lytic or lysogenic. Only lytic strains and those that are not the last lytic mutants of lysogenic phages should be used for therapeutic purposes [12]. The range of hosts that can be infected by a phage is important for phage therapy, because it determines the potential number of strains which can be treated, and hence the effectiveness of phage therapy [13]. One of the features of phage therapy is that most phages are able to infect a very narrow range of hosts. However, lytic staphylococcal phages have been found to have a wide range of hosts [14,15] and are able to infect coagulase-positive and coagulase-negative staphylococci of animal and human origin [16]. The number of hosts can be limited by active host resistance mechanisms, such as clustered regularly interspaced short palindromic repeats, or modification and restriction systems (PM), which actively suppress phage infection, or passive mechanisms, such as loss of receptors on the surface of bacteria for phage adsorption. They may occur due to specific adaptation against phage infection or be by-products of selection against other stresses. However, there are specific phage mechanisms that counteract host resistance and help expand the range of phage hosts [14,17]. Therefore, important components of effective phage therapy are the identification of bacteriophage-sensitive microorganisms that are pathogens, including *S. aureus*.

The aim of this work was to determine the sensitivity of *S. aureus* cultures of different biological origins to commercially available bacteriophages and phages of *S. aureus* var*. bovis*, which has been isolated on dairy farms, to create a drug for the treatment of mastitis in cows.

## Materials and Methods

### Ethical approval

Ethical approval was not necessary for this study.

### Study period and location

Samples of cows’ secretions with signs of mastitis and sewage water were used as the study material and were taken during 2014 - 2020 years. A working set of microorganisms from field strains isolated on dairy farms in Ukraine have been created as hosts for the cultivation of phages active against *S. aureus* *var*. *bovis*. Laboratory analysis was carried out in the Department of Infectious and Parasitic Diseases, State Agrarian and Engineering University in Podilya, Ukraine.

### Strains of bacteria and phages

We used cultures of *S. aureus* isolated from the secretions of the mammary glands of cows with signs of mastitis (96 strains), cultures isolated from home-made dairy products sold in agri-food markets (26 strains), cultures isolated from human habitats with various inflammatory processes (74 strains), and archival strains of *S. aureus* No.209-P and *S. aureus* (ATCC 25923). Previously, the biotype of *S. aureus* in culture has been determined by assessing the pigment color, presence of beta-hemolysis, coagulase activity in bovine plasma, and growth on medium with crystalline violet [18]. Other types of staphylococci used in the study were isolated on dairy farms and identified using the STAPHY-test 24 (Erba Lachema, Brno, Czech Republic).

The bacteriophages *Phage SAvB07*, *Phage SAvB08*, *Phage SAvB12*, and *Phage SAvB14* were previously isolated and described by us [6]. We also used drugs based on commercially available bacteriophages: Staphylococcal bacteriophage (Microgen, Russia) and Intestifag (Pharmex Group, Boryspil, Ukraine).

### Determination of lytic activity of phage drug

Determination of the range of action of bacteriophages on the clinical isolates of staphylococci was carried out the drip method. In this method, 3-4 drops of 18-24 h broth culture of the staphylococci were pipetted onto the surface of BD Baird-Parker Agar (HiMedia, Mumbai, India) in Petri dishes. The optical density of the control inoculum was 0.5 McFarland units, as measured using a densitometer, corresponding to 1.5×10^8^ microbial cells/ml. The cultures were evenly distributed on the surface of the medium using a sterile spatula. Dishes with seeded media were dried in an incubator at 25°C for 15-20 min. A drop of the test drug was then applied to the surface of the plate, which was tilted to distribute the drug evenly. The plates were then incubated at a temperature of 37°C, and the results were evaluated after 18-24 h. As a control, sterile nutrient broth was applied to the surface of the culture medium.

The degree of lysis was assessed as follows. The symbol “++++” indicated confluent lysis indicated by lack of culture growth; “+++” indicated semi-confluent lysis, with some growth of culture in the lysis zone, resulting in the growth of several colonies; “++” indicated the presence of more than 50 colonies of phage (lysis spots) at the site of application of a drop of phage; “+” indicated the presence of from 20 to 50 colonies of phage at the site of application of a drop of phage; “+/−” indicated the presence of less than 20 colonies of phage at the site of application of a drop of phage; and - indicated complete absence of lysis. Results from “+++” to “++++” were considered to be positive reactions. The study was performed in triplicate.

### Statistical analysis

Statistical processing of the results was carried out using the program Statistica 6.0. (StatSoft Inc., USA). Non-parametric analytical methods were used (Wilcoxon–Mann–Whitney criteria). The arithmetic mean (x) and the standard error of the mean were determined. Differences between values were considered significant at p<0.05.

## Results

We initially determined the effectiveness of commercially available bacteriophages. Table-1 shows the results of the study of the effects of the bacteriophage drugs containing staphylococcal phages, staphylococcal bacteriophage, and Intestifag, on cultures of *S. aureus*, isolated from different habitats.

**Table-1:** Sensitivity of *Staphylococcus aureus* of different origin to bacteriophage drugs, %.

Origin of strains	Number of studied cultures, n	Studied drugs	

		“Staphylococcal bacteriophage”	“Intestifag”
Cultures isolated from the secretion of the mammary gland of cows with signs of mastitis	96	0	0
Cultures isolated from dairy products sold on agri-food markets	26	0	0
Cultures isolated from human habitats with different inflammatory processes	74	25.6±2.5	91.8±8.2
Archival strains: *S. aureus* No.209-P, *S. aureus* (ATCC 25923)	2	0	100

Table-1 shows that the drug Intestifag was the most active against isolated staphylococci and archival strains. It lysed cultures isolated from human habitats and archival strains of *S. aureus* No.209-P and *S. aureus* (ATCC 25923) in 91.8-100% of cases. The drug staphylococcal bacteriophage destroyed only 25.6% of cultures of *S. aureus* isolated from humans, 3.6 times less than Intestifag. Staphylococcal bacteriophage did not affect the growth of museum strains. Neither commercial drug lysed the cultures isolated from the secretions of the mammary glands of cows nor cultures isolated from dairy products sold in agri-food markets.

Table-2 shows the sensitivity of *S. aureus* of different origins to bacteriophages which were isolated by us on dairy farms. The data, given in Table-2, indicate that the bacteriophages *Phage SAvB14*, *Phage SAvB12*, *Phage SAvB08*, and *Phage SAvB07*, which were isolated on dairy farms, are characterized by a specificity of lytic action against staphylococci isolated from different habitats. Thus, the largest number of sensitive staphylococcal cells was observed under the action of the bacteriophage *Phage SAvB14*. This phage acted lytically on 92.7±8.3% of *S. aureus* strains isolated from the secretions of the mammary glands of cows with signs of mastitis and 69.2±6.4% of cultures isolated from dairy products sold in agri-food markets. However, *Phage SAvB14* showed active lytic action against human staphylococci only in 35.1±3.1% of cases and did not affect the archival strains. Other bacteriophages studied were less active against staphylococci isolated from different habitats. In particular, *Phage SAvB12*, *Phage SAvB08*, and *Phage SAvB07* lysed 1.2-1.7 times fewer cultures isolated from the mammary gland of cows and 6-18 times fewer cultures isolated from dairy products, than *Phage SAvB14*. A slight anti-staphylococcal effect was observed in *Phage SAvB12*, which lysed 16.2±1.3% of cultures isolated from human habitats. The phages *Phage SAvB12*, *Phage SAvB08*, and *Phage SAvB07*, also isolated on dairy farms, did not show activity against archival strains of *S. aureus No.209-P*, *S. aureus* (ATCC 25923), or *S. aureus* isolated from human habitats. The etiology of mastitis in cows involves the well-known pathogen *S. aureu*s and other species of the genus *Staphylococcu*s. We investigated the lytic activity of isolated phages against cultures of *Staphylococcus* spp. isolated on dairy farms (Table-3).

**Table-2:** Sensitivity of *S. aureus* of different origin to bacteriophages isolated on dairy farms, %.

Origin of strains	Number of studied cultures, n	Studied drugs			

		*Phage SAvB14*	*Phage SAvB12*	*Phage SAvB08*	*Phage SAvB07*
Cultures isolated from the secretion of the mammary gland of cows with signs of mastitis	96	92.7±8.3	75.0±6.7	71.8±5.7	54.2±4.3
Cultures isolated from dairy products sold on agri-food markets	26	69.2±6.4	11.5±1.0	3.8±0.3	7.7±0.6
Cultures isolated from human habitats with different inflammatory processes	74	35.1±3.1	16.2±1.3	0	0
Archival strains: *S. aureus* No.209-P, *S. aureus* (ATCC 25923)	2	0	0	0	0

*S. aureus=Staphylococcus aureus*

The data, given in Table-3, show that the bacteriophages studied are capable of infecting not only *S. aureus* but also other species of staphylococci. The most lytically active of the isolated phages was *Phage SAvB14* which lysed from 41.5% to 62.1% of the identified cultures of coagulase-negative staphylococci. In species such as *Staphylococcus saprophyticus* and *Staphylococcus xylosus*, on average around 60% of cells were sensitive to *Phage SAvB14. Phage SAvB1*2 was the least lytic against coagulase-negative staphylococci. Species such as *Staphylococcus epidermidi*s, *Staphylococcus haemolyticus*, an*d S. saprophyticus* were not lysed by this phage. However, 65.2±5.8% of *S. xylosu*s cultures were sensitive to this phage.*Phage SAvB08* and *Phage SAvB0*7 had virtually no effect on *S. epidermidi*s or *S. haemolyticu*s. *Phage SAvB0*8 lysed 48.2±4.3% of cultures of *S. saprophyticu*s and 39.1±3.1% of *S. xylosu*s. *Phage SAvB0*7 lysed these staphylococcal species in 36.2±3.2% and 69.5±5.5% of cases, respectively.

**Table-3:** Sensitivity of *Staphylococcus* spp. to bacteriophages isolated on dairy farms, %.

Type of staphylococci	Number of studied cultures	Studied bacteriophages			

		*Phage SAvB14*	*Phage SAvB12*	*Phage SAvB08*	*Phage SAvB07*
*Staphylococcus epidermidis*	39	48.7±4.3	0	20.5±1.6	0
*Staphylococcus haemolyticus*	41	41.5±3.3	0	0	0
*Staphylococcus saprophyticus*	58	62.1±4.8	0	48.2±4.3	36.2±3.2
*Staphylococcus xylosus*	23	60.8±4.8	65.2±5.8	39.1±3.1	69.5±5.5

## Discussion

Interest in lytic bacteriophages as antimicrobial agents is growing, due to their ability to self-replicate, their ubiquity in nature, and their high specificity to target bacteria. Phages which have high lytic activity and a wide range of hosts are therapeutically promising [6,12,19]. This study was performed to determine the lytic properties and to evaluate the range of hosts of bacteriophages of *S. aureus* var*. bovis*, which were isolated on dairy farms. These phages may be used to create a drug for the treatment of mastitis in cows.

To produce an objective assessment of the effectiveness of the isolated bacteriophages, we determined the susceptibility of *S. aureus* isolated from different habitats to existing bacteriophage drugs, which are recommended for use in staphylococcal infections. The drugs, which are based on commercially available bacteriophages, were ineffective against cultures of *S. aureus* isolated from dairy products and from cows with mastitis. This observation indicates that the hosts of the bacteriophage strains used in the manufacture of drugs do not include strains of *S. aureus* var*. bovis*.

We obtained almost opposite data when testing bacteriophages isolated on dairy farms. Thus, all our studied bacteriophages – *Phage SAvB14, Phage SAvB12, Phage SAvB08*, and *Phage SAvB07* – to some extent *S. aureus* isolated from the secretions of the mammary glands of cows with signs of mastitis, and cultures isolated from dairy products sold in agri-food markets, and were ineffective against strains from human habitats. *Phage SAvB14 (*69.2±6.4-92.7±8.3%) had the largest circle of hosts among the studied bacteriophages. Therefore, this strain can be recommended as an antimicrobial agent with which to create a drug for the treatment of staphylococcal mastitis in cows.

Similar results have been observed by other researchers [11,20]. Thus, the rates of infection of isolates of *S. aureus* var*. hominis* by phage *fRuSau02* were significantly higher than those of staphylococci derived from animals [11]. Phage *ISP* has also effectively infected isolates of staphylococci isolated in hospitals, but has been unable to infect *S. aureus* strains isolated from pigs [20]. The stability of strains of different biological origins may be due to minor structural differences in teichoic acid [9]. It is also likely that some strains can develop phage resistance without modification of phage receptors. Strains isolated from animals often belong to different clonal complexes, which explain their different phage profiles [9].

The weak lytic activity of the other phages studied, *Phage SAvB12, Phage SAvB08*, and *Phage SAvB07*, may indicate their lysogenicity. This may be of concern when they are used as therapeutic agents, due to the potential for integration of the prophage into the host genome without causing lysis. Some virulence factors of *S. aureus* are encoded by staphylococcal prophages. However, it has been shown that lysogenic staphylococcal phages can also be used as antimicrobial drugs to combat MRSA, but with mutated lysogeny modules [21]. The combination of different domains with different cell wall recognition properties often leads to synergies and opens up promising areas for their use as antibacterial agents.

Researchers also report a wide range of activity among staphylococcal endolysins, the cell wall binding domain of which recognizes different types of staphylococci [22,23]. Our studies show that specific phages of *S. aureus* var*. bovis* can infect staphylococcal species such as *S. epidermidis*, *S. haemolyticus*, *S. saprophyticus*, and *S. xylosus*. The widest range of hosts was found in *Phage SAvB14*, which may indicate its polyvalence. The wide range of activity can be explained by the cleavage sites of cysteine/histidine-dependent amidohydrolase/peptidase and amidase domains of staphylococcal endolysins, which are conserved in the peptidoglycans of *S. aureus* and other staphylococcal species.

All of the staphylococcal bacteriophages studied multiplied more effectively in *S. xylosus* cells than in other species of coagulase-negative staphylococci. Similar data have been obtained [24,25] in a study of the ranges of hosts of wild-type phages *Team1*, *phi812*, which revealed that phages that reproduce on *S. xylosus* were able to lyse 52 of 57 different strains of *S. aureus*.

This study complements our earlier data on laboratory studies of phages of *S. aureus* var*. bovis*, including their morphology, lytic activity, and the effect of *Phage SAvB14* on staphylococcal biofilms [3,6,26]. Prototypes of a drug based on *Phage SAvB14* have been created, and clinical trials are underway.

## Conclusion

These studies indicate that effective phage therapy requires consideration of the biological origin of staphylococcal strains and, accordingly, the use of bacteriophages that are specific to their hosts. *Phage SAvB14* has the widest range of hosts among the bacteriophages isolated on dairy farms, which makes it the best candidate for the development of a phage drug for the treatment of mastitis in cows. As an additional host for its replication, it is possible to use *S. xylosus*, which is non-pathogenic.

## Authors’ Contributions

YH, MK, and SK: Conceived and executed the idea, designed experiments, analyzed results, and revision of the manuscript. SL, SP, and NB: Collected samples, performed experiments, contributed in implementation of the research. All authors read and approved the final manuscript.
